# Brain Gene Silencing with Cationic Amino-Capped Poly(ethylene glycol) Polyplexes

**DOI:** 10.3390/biomedicines10092182

**Published:** 2022-09-03

**Authors:** Abdullah A. Alamoudi, Paula A. Méndez, David Workman, Andreas G. Schätzlein, Ijeoma F. Uchegbu

**Affiliations:** 1 UCL School of Pharmacy, 29-39 Brunswick Square, London WC1N 1AX, UK; 2 Department of Pharmaceutics, Faculty of Pharmacy, King Abdulaziz University, Jeddah 21589, Saudi Arabia; 3 Materials Science Research Group, Institute of Chemistry, Faculty of Exact and Natural Sciences, University of Antioquia, Calle 70 N° 52-21, A.A 1226, Medellín 050010, Colombia; 4 Nanomerics Ltd., 2 London Wall Place, London EC2Y 5AU, UK

**Keywords:** siRNA delivery, brain, gene silencing, polyethylene glycol (PEG), nanoparticles, intranasal

## Abstract

Therapeutic gene silencing in the brain is usually achieved using highly invasive intracranial administration methods and/or comparatively toxic vectors. In this work, we use a relatively biocompatible vector: poly(ethylene glycol) star-shaped polymer capped with amine groups (4APPA) via the nose to brain route. 4APPA complexes anti- itchy E3 ubiquitin protein ligase (anti-ITCH) siRNA to form positively charged (zeta potential +15 ± 5 mV) 150 nm nanoparticles. The siRNA-4APPA polyplexes demonstrated low cellular toxicity (IC50 = 13.92 ± 6 mg mL^−1^) in the A431 cell line and were three orders of magnitude less toxic than Lipofectamine 2000 (IC50 = 0.033 ± 0.04 mg mL^−1^) in this cell line. Cell association and uptake of fluorescently labelled siRNA bound to siRNA-4APPA nanoparticles was demonstrated using fluorescent activated cell sorting (FACS) and confocal laser scanning microscopy (CLSM), respectively. Gene silencing of the ITCH gene was observed in vitro in the A431 cell line (65% down regulation when compared to the use of anti-ITCH siRNA alone). On intranasal dosing with fluorescently labelled siRNA-4APPA polyplexes, fluorescence was seen in the cells of the olfactory bulb, cerebral cortex and mid-brain regions. Finally, down regulation of ITCH was seen in the brain cells (54 ± 13% ITCH remaining compared to untreated controls) in a healthy rat model, following intranasal dosing of siRNA-4APPA nanoparticles (0.15 mg kg^−1^ siRNA twice daily for 3 days). Gene silencing in the brain may be achieved by intranasal administration of siRNA- poly(ethylene glycol) based polyplexes.

## 1. Introduction

The recent approval of both mRNA vaccines [1] and gene silencing therapeutics [2] has demonstrated that nucleic acid based therapeutics may be exploited for a wide variety of applications. The advances made with the COVID-19 vaccines will undoubtedly usher in a new generation of nucleic acid therapeutics. One area that remains to be tackled and solutions offered is nucleic acid therapies for central nervous system diseases. Onasemnogene Abeparvovec-xioi, indicated for the treatment of spinal muscular atrophy, was the first gene therapeutic to be approved for the treatment of a neurological condition [3]. In this therapeutic an adeno-associated virus (AAV), specifically AAV9, delivers the survival motor neuron 1 (SMN1) gene to produce SMN protein, providing continuous protein expression and therapeutic efficacy in paediatric patients following an intravenous infusion. Recent evidence of brain gene delivery in animals’ gyrencephalic brains has emerged following intraarterial delivery of alpha mannosidase in the AAVhu.32 vector to a cat model of the lysosomal storage disease alpha mannosidosis [4]; with animals showing a reduction in the severity of neurological signs and extended life spans, when compared to untreated controls. With respect to brain gene silencing, the intracranial administration of siRNA targeting the BCL2 gene in a peptide nanofibre vector, hydrophobically modified siRNAs targeting the luciferase gene or hydrophobically modified siRNA targeting the huntingtin gene all resulted in a degree of gene silencing [5,6,7]. A further technique that has been tried is the transient disruption of the blood brain barrier by the intracarotid administration of 25% mannitol and hydrophobically modified siRNA targeting the huntingtin gene, with this strategy also resulting in gene silencing [8].

Nose to brain drug delivery is possible in humans, as evidenced by the use of insulin and oxytocin solutions [9,10] and the intranasal delivery of peptide nanoparticles to rats produces a centrally mediated therapeutic response [11]. The use of nanoparticles to deliver siRNA to the olfactory bulb and brain via the nasal route has also been reported in mice [12,13] and rats [14,15]. We have previously shown neuronal delivery of siRNA in the olfactory bulb tissue of rats following intranasal dosing; however, we did not show delivery to other areas of the brain or establish gene silencing in vivo in that study [15]. Rodriguez et al. report using the comparatively toxic poly(ethylenimine) (IC50 = 1.9 μg mL^−1^ in the A431 cell line and IC50 = 5.2 μg mL^−1^ in the A549 cell line) [16] to deliver siRNA intranasally to mice in order to silence the mice Beclin1 gene [12]; reducing gene expression by between 43 and 65% following the administration of 16 μg siRNA per mouse. Kim et al. use a polyamidoamine dendrimer (surface modified with arginine residues) derivative as a vector to deliver siRNA to silence the high mobility group box 1 (HMGB1) gene in rats [14]; reducing the target protein levels by about 40%. This polyamidoamine dendrimer (ePAM-R) showed signs of cytotoxicity (80% cell viability) at a concentration of 50 μg mL^−1^ [17]. Finally, Sava et al. report on the use of mangafodipir-chitosan siRNA nanoparticles for the gene silencing of the huntingtin gene following intranasal delivery; with the siRNA hydrophobically modified with cholesterol [13]. Mangafodipir, a manganese chelate used as a magnetic resonance contrast agent [18], was used as a polymer cross linking agent to stabilise the polyplexes [13]. The formulation reduced huntingtin protein levels by about 50% when compared to controls. It should be noted, however, that mangafodipir is a known teratogen [19].

We are motivated to study the problem of delivering brain gene silencing as there are no clear solutions at present that adequately address this problem. The literature reveals that experimental gene silencing in preclinical studies is currently achievable using intracranial methods and either hydrophobised siRNAs or siRNA complexed particles [5,6,7]. For the non-invasive intranasal method of delivering siRNA to the brain this usually involves the use of hydrophobised siRNA and the use of siRNA complexed particles, with carriers made from polymers, polymer—metal cation chelates and dendrimers, all of questionable biocompatibility [12,13,14,15]. We assert that the early promise of intranasal gene silencing could advance further into clinical development, if carriers with a reduced potential toxicity are discovered. This reduced toxicity is especially important, as the intranasal carriers must cross healthy brain tissue to arrive at the target. In an effort to introduce more biocompatible carriers for the treatment of chronic neurological disorders using brain gene silencing via the nose to brain route, here, we introduce a comparatively low molecular weight star shaped poly(ethylene glycol) derivative with good cell biocompatibility, as a carrier for siRNA. We demonstrate good cell biocompatibility, siRNA distribution of intranasal siRNA to the olfactory bulb, frontal cortex and mid-brain and gene silencing in the olfactory bulb and brain tissue, using this new carrier.

## 2. Material and Methods

### 2.1. Materials

All chemicals and reagents were of reagent grade and were supplied by Sigma Aldrich, Poole, UK, unless otherwise indicated. 4APEG-NH_2_ [Tetra-O,O,O,O-poly(ethyleneglycol-O-2- ethyleneimine), 2000 Da] was supplied by Jenkem Technology, Plano, TX, USA. Triethylamine (TEA) and anhydrous sodium sulphate were supplied by Fisher scientific, Loughborough, UK. Hydrochloric acid (HCl) was supplied by VWR International, Poole, UK. Deuterium oxide (D_2_O) was supplied from Goss Scientific, Crewe, UK, and dialysis membrane (molecular weight cut off = 1 kDa) was supplied from Spectrum Chemical Manufacturing Corporation, New Brunswick, NJ, USA.

### 2.2. Synthesis of Tetra-O,O,O,O-[Poly(Ethyleneglycol-O-2-Ethyleneimine)-Graft-N-(3-Propylamine)-]-Pentaerythritol (4APPA)

**Figure 1:**
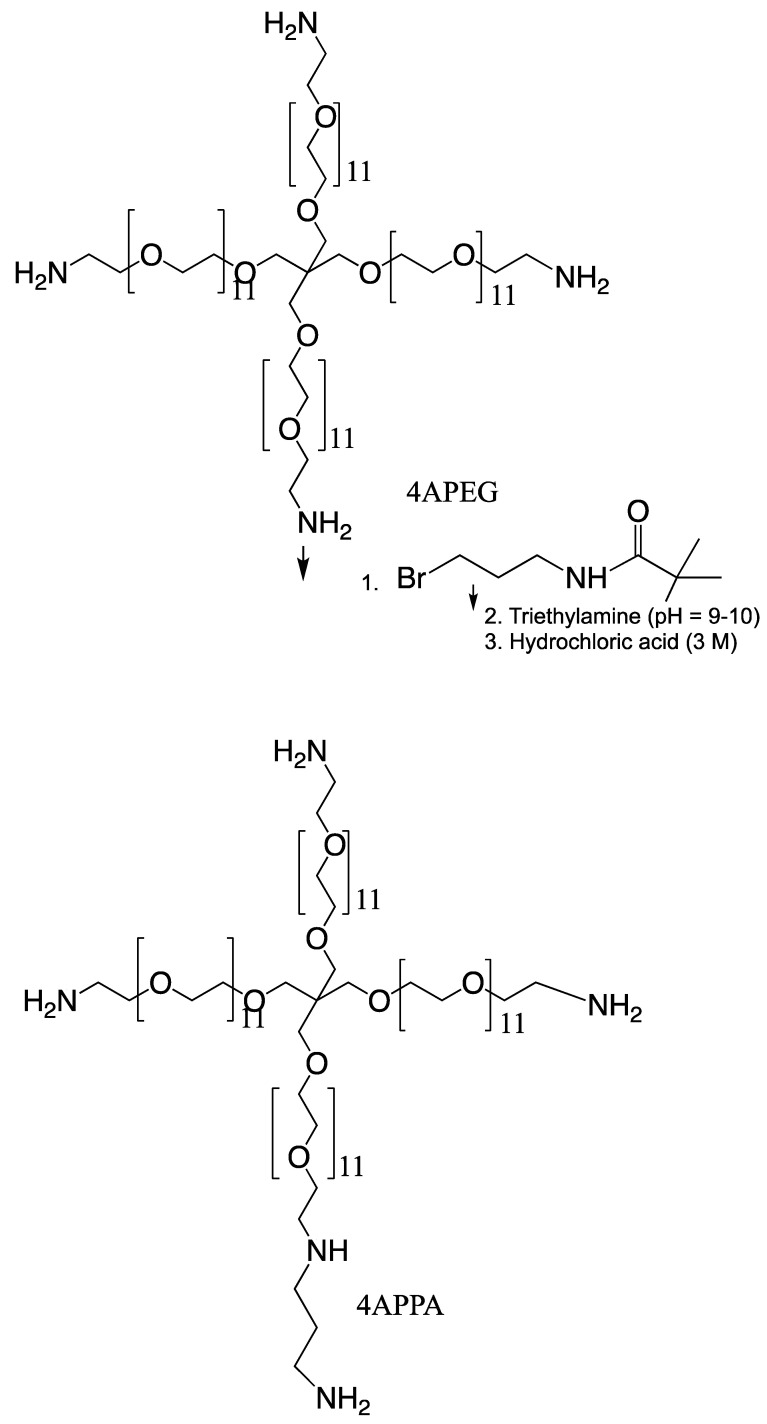
Synthesis of 4APPA.

Synthesis of tetra-O,O,O,O-[poly(ethyleneglycol-O-2-ethyleneimine)-graft-N-(3-propylamine)-]-pentaerythritol (4APPA) was carried out by reacting tetra-O,O,O,O-poly(ethyleneglycol-O-2- ethyleneimine) (4APEG-NH_2_) and 3-(Boc-amino) propyl bromide (3-Boc-APBr), in a nucleophilic substitution reaction, followed by deprotection (Figure 1). 4APEG-NH_2_ (0.6324 g, 0.316 mmol) was dissolved in chloroform (37.5 mL) and 3-Boc-APBr (1.26490 g, 5.31 mmol) was dissolved in chloroform (25 mL). The 3-Boc-APBr chloroform solution was added to the 4APEG-NH_2_ chloroform solution with continuous stirring. Triethylamine (TEA, 6.25 mL, 44.84 mmol) was then added to this mixture and the reaction refluxed for 51 h at 60 °C. At the end of the reaction, the solvent was removed by evaporation under reduced pressure at 40 °C and the crude product was obtained as a yellow paste.

To the crude product was carefully added HCl (3 M, 125 mL) in order to effect deprotection of the Boc group. The resulting solution was left stirring at room temperature for 90 min. The deprotected product in solution was then dialysed against water (5 L) using a dialysis membrane with a 1 kDa cut off, for 23 h with 5 changes of dialysis medium. The dialysate was lyophilised and the dried yellow paste was reconstituted in water (6 mL). The reconstituted dialysate was neutralised with sodium hydroxide (NaOH) powder (final solution pH = 10). This solution was extracted with two volumes of dichloromethane. The extraction was performed twice, and the organic phase separated and evaporated under reduced pressure at 33 °C until a yellow paste was achieved. The sample was reconstituted in MilliQ water, freeze dried and analysed by proton nuclear magnetic resonance spectrometry (20 mg mL^−1^ in D_2_O, ^1^H NMR, Bruker Avance 400 MHz spectrometer, Bruker Instruments, Coventry, UK) and matrix assisted laser desorption time of flight spectrometry (MALDI-TOF, Voyager-DE™ PRO Biospectrometry™ Workstation, PerSeptive Biosystems Inc., Framingham, MA, USA). For MALDI-TOF analysis, 2,5-dihydroxy benzoic acid (10 mg mL^−1^) in acetonitrile, water, trifluoroacetic acid (50:50:0.1) was used as a matrix and mixed at a 1:1 weight ratio with the sample (4APPA). Data were collected and processed using the Voyager Biospectrometry™ Workstation.

4APPA ^1^H NMR (D_2_O): δ = 1.81 (m, 2H, -NH-CH_2_**-CH_2_**-CH_2_-NH_2_), 2.76 (t, 2H, -CH_2_**-**CH_2_**-**NH**-CH_2_-**CH_2_-CH_2_-), 2.87 (t, 2H, -CH_2_-CH_2_**-CH_2_-**NH_2_), 2.94 (t, 6H, -O-CH_2_**-CH_2_-**NH**-**CH_2_**-**CH_2_-CH_2_-), 3.55 (s, 10H, -C-**CH_2_**-O-), 3.65–3.8 (b, 208H, -O-**CH_2_-CH_2_**-O-**CH_2_-**CH_2_-NH-). Yield = 0.19 g (30%). MALDI analysis ms (main peak) m/z calculated for C_105_H_220_N_8_O_44_ = 2298.3 Da, found 2164.35 Da. Degree of propylamine substitution (obtained by comparing the methylene propylamine protons (-NH-CH_2_-**CH_2_**-CH_2_-N-) at 1.88 pm with the methylene pentaerythritol protons (-C-**CH_2_**-O-) at 3.55 ppm: 21 mole%, approximately 1 propylamine group per molecule.

The degree of propylamine substitution (obtained by comparing the shift in the molecular weight distribution of 4APPA with that of the starting material (4APEG-NH_2_, molecular weight = 2053.9 Da)) = 48%; this value was considered to be less accurate due to the broad distribution of the MALDI-TOF peak. For example, there is a difference in the determined m/z data for 4APEG-NH_2_ and the expected data (please see below). ^1^H NMR data was used to compute the actual level of propylamine substitution.

4APEG-NH_2_ ^1^H NMR (D_2_O): δ = 2.8 (t, 2H, -O-CH_2_**-CH_2_-**NH_2_), 3.4 (s, 2H, -C-**CH_2_**-O-), 3.56 (t, 2H, -O**-CH_2_-**CH_2_-NH-), 3.6–3.75 (b, 40H, -O-**CH_2_-CH_2_**-O-). MALDI analysis ms (main peak) m/z calculated for C_93_H_192_N_4_O_44_ = 2070.5 Da, found 2053.9 Da.

### 2.3. Polyplex Formulations

4APPA-siRNA complexes were prepared at different nitrogen (from 4APPA) to phosphate (from siRNA) ratios (N, P ratios = 0.5, 2, 21.5, 32, 43, 53.5 and 107).

Anti-itchy E3 ubiquitin protein ligase (ITCH-siRNA [5′ CCACAACACACGAAUUACA-3′]) was from Eurofins MWG, Operon, UK. The ITCH-siRNA powder was dissolved in a 5× concentrated siMAX dilution buffer (30 mM HEPES, 100 mM KCl, 1 mM MgCl_2_, pH = 7.3, Eurofins MWG) to a final concentration of either 1 mg mL^−1^ or 2 mg mL^−1^ depending on experimental requirements.

4APPA—ITCH siRNA complexes were prepared by adding ITCH-siRNA solution (1 mg mL^−1^, 0.01 mL) in sterile phosphate buffer (2 mM, pH = 6.0) to appropriate volumes of 4APPA polymer stock solution (4 mg mL^−1^) in sterile phosphate buffer (2 mM, pH = 6.0) to a final siRNA concentration of 20 μg mL^−1^ and a final formulation volume of 500 μL. The siRNA and 4APPA solutions were gently mixed together using a pipette and incubated at room temperature for 1 h to ensure complex formation.

The resulting complexes (pH = 6.0) were sized and their zeta potential measured without dilution (Malvern Zetasizer 3000HS, Malvern, UK). The size distribution was measured in disposable cuvettes (Malvern Instruments, Malvern, UK) and the zeta potential measured in disposable zeta potential cuvettes (Malvern Instruments, Malvern, UK). Sizing and zeta potential measurements were conducted on 5 independent samples and each sample was measured three times. Prior to analyses, 96 nm latex nanoparticles in phosphate buffer (2 mM, pH = 6.0) were sized and the zeta potential of a zeta potential standard (zeta potential = −42 mV) was analysed. All size and zeta potential data from these standards were in accordance with the manufacturers’ specifications.

The complexes were also imaged using a scanning electron microscope (SEM) (FEI Quanta 200 FEG ESEM, FEI, Hillsboro, OR, USA). A 20 μL sample was placed on an SEM nickel grid. Samples were desiccated for at least 48 h to ensure complete drying. The dried mounted sample was coated with an ultrathin coating of electrically conducting gold by high-vacuum evaporation and imaged.

### 2.4. In Vitro Studies

#### 2.4.1. Cell Cytotoxicity

A431 cells (ATCC CR L-1555) were seeded in 96 well plates at a concentration of 5000 cells per well (100 μL) in Modified Eagle Medium (MEM) supplemented with foetal bovine serum (FBS, 10% *v*/*v*) and glutamine (2 mM). To each well was added an aliquot of the 4APPA solution (50 μL) obtained by diluting a stock solution of 4APPA (100 mg mL^−1^). To each well was then added 50 μL of MEM. The cells were incubated with the 4APPA for 6 hr at 37 °C in 5% CO_2_. At the end of the incubation time, cells were washed twice with phosphate buffered saline (PBS, pH = 7.4), ensuring removal of the polymer. The cells were then replenished with fresh MEM (200 μL) and incubated for a further 48 h post-treatment, with daily changes of the medium. Cells were washed twice with PBS and the MTT reagent [0.5 mg mL^−1^, 50 μL, (3-(4,5-dimethylthiazol-2-yl)-2,5-diphenyl tetrazolium bromide] in fresh medium added to each well. After incubation for 3 h at 37 °C, the supernatant was removed, the cells lysed with dimethylsulphoxide (DMSO, 200 μL) and the absorption of the supernatant (obtained by centrifugation, Eppendorf Centrifuge 5430 R, Eppendorf UK, Stevenage, UK) measured at 570 nm. Triton X-100 was used as a positive control, while untreated cells acted as negative controls. Lipofectamine^®^ 2000, a commercial transfection reagent, was used as a comparative control (by diluting a stock of the transfection reagent −0.1 mg mL^−1^). Cell survival rates were expressed as a percentage of untreated controls and IC50 values recorded.

#### 2.4.2. In-Vitro Gene Silencing Efficiency

The A431 cell line (ATTC, CRL-1555TM) was chosen for transfection experiments due to its high expression of ITCH. T25 cm^2^ flasks were seeded with 400,000 cells per flask and incubated overnight. Suspensions of 4APPA-ITCH-siRNA (with nitrogen to phosphate ratios of 21.5, 32, 43 and 53.5, 10 μg siRNA-ITCH, 500 μL) in phosphate buffer (2 mM, pH = 6) were added to the flasks and the cells incubated for 6 h. Lipofectamine^®^ 2000 (30 μL) was complexed with ITCH-siRNA at a final ITCH-siRNA dose of (3 μg) as per the manufacturer’s protocol and used as the positive control. Lower doses of siRNA were used in the Lipofectamine formulations due to the toxicity of Lipofectamine and the concentrations used were in accordance with the manufacturer’s instructions. Non-treated cells, ITCH-siRNA alone and 4APPA—scrambled siRNA polyplexes were used as negative controls. At the end of the incubation period, the media was removed and replaced with fresh MEM containing FBS (10% *w*/*v*). The cells were left incubating for a further 48 h, with media being replaced daily. Gene silencing was then analysed using a Western Blotting assay.

At the end of the gene silencing experiment, cells were harvested and lysed in radioimmunoprecipitation buffer (RIPA, 0.02 mL) containing ThermoFisher Scientific Halt Protease Inhibitor Cocktail (protease inhibitors, phosphatases and EDTA, at a ratio of 1 mL RIPA buffer to 0.01 mL protease inhibitor cocktail, ThermoFisher Scientific, Hempstead, UK). The resulting lysates were centrifuged at 14,400 rpm for 15 min at 4 °C and the protein content of the supernatant determined using a micro bicinchoninic acid (BCA) assay kit according to the manufacturers protocol (ThermoFisher Scientific, Hempstead, UK). An aliquot of the supernatant containing 30 μg protein was analysed on an SDS-PAGE gel (sodium dodecyl sulphate-polyacrylamide gel, NuPAGE Bis-Tris gel) by electrophoresis at 200 V for 50 min. The gel was transferred to a nitrocellulose membrane and blocked with blocking reagent (5% *w*/*v* skimmed milk in PBS—pH = 7.4-containing 0.1% *v*/*v* Tween 20) overnight at 4 °C to prevent antibody non-specific binding. The membrane was washed five times over 30 min, three times with PBS containing Tween 20 (0.1% *v*/*v*, pH = 7.4), followed by two washes with PBS (pH = 7.4). Immunostaining of the membrane then took place, first with the primary antibodies of both anti-Actin protein (the house-keeping gene, 0.2 mg mL^−1^ in 4 μL) and anti-ITCH protein (the target gene, 0.25 mg mL^−1^ in 20 μL) all diluted in Tween 20 (0.1% *v*/*v* in PBS, 10 mL) and incubated with the membrane for 2 hrs at room temperature. The membrane was rinsed three times with PBS containing Tween 20 (0.1% *v*/*v*, pH = 7.4) for 5 min each time followed by a further two rinsing with PBS (pH = 7.4) for 5 min each time. This was followed by incubation for 1 h at room temperature with a secondary antibody conjugated to horseradish peroxidase (1 mg mL^−1^, 10 μL) diluted in Tween 20 (0.1% *v*/*v* in PBS, 10 mL). The five washing steps described above were repeated to remove labelling reagent residues and the blot was developed with a SuperSignal westPICO chemiluminescent detection kit as per the manufacturer’s instructions (ThermoFisher Scientific, Hempstead, UK). Membranes were imaged using a UV camera.

Data were generated as gel blot images. Furthermore, the ITCH relative expression levels were normalised to the reference protein actin to correct for sample variation. The ratio of ITCH expression to actin expression was determined and ITCH expression was compared between samples. ITCH protein levels were expressed as down-regulation percentages by measuring the signal optical density (Western blot densitometry analysis) using a UV camera (Gel Doc XR+, Bio-Rad Laboratories, Watford, UK).

#### 2.4.3. siRNA Cell Uptake

To investigate 4APPA-siRNA nanoparticle cellular uptake and intracellular trafficking, a fluorescently labelled siRNA was complexed to 4APPA, the complex applied to cells and the cells analysed using both fluorescence activated cell sorting (FACS) and confocal laser scanning microscopy (CLSM).

For FACS analyses, a 6-well plate was seeded with 300,000 cells per well and incubated overnight. Following this incubation period, each well was washed with D-PBS (Dulbecco’s Phosphate Buffered Saline, 5 mL, pH = 7.1) and then MEM (devoid of FBS) was added (1.5 mL) to each well. Complexes of 4APPA and FAM^TM^ labelled siRNA (fluorescein amidite labelled siRNA, excitation λ = 494 nm, emission λ = 520 nm, ThermoFisher Scientific, Hempstead, UK) at a nitrogen to phosphate ratio of 32 were prepared in phosphate buffer (2 mM, pH = 6) and in a final volume of a 500 μL dispersion containing 10 μg siRNA. This polyplex dispersion was added to each cell well and the cells incubated for 6 hrs. Lipofectamine 2000^TM^ (30 μL) was complexed with FAM^TM^ labelled siRNA at a final FAM^TM^-siRNA content of 3 μg in a 30 μL volume, using the manufacturer’s instructions. Lipofectamine 2000^TM^ formulation was used as a positive control. Non-treated cells and cells treated with FAM^TM^-siRNA alone were used as negative controls. After incubation, the medium was removed and cells washed three times to ensure all formulations had been removed. Cells were detached by trypsinization as described previously. Trypsinization involved aspirating the media, washing cells with PBS—ethylenediamine tetraacetic acid (PBS-EDTA, 10 mL, 0.46 mg mL^−1^ EDTA, pH = 7.4), adding trypsin—EDTA (0.25% *w*/*v* trypsin and 0.38 mg mL^−1^ EDTA, 3 mL) and incubating for 3 min. Trypsinization was stopped by adding MEM (7 mL) and the cells centrifuged (3000 rpm for 3 min at 4 °C). Cells were re-suspended in complete medium (10 mL) and an aliquot (3 mL in MEM) analysed by fluorescence activated cell sorting (MACSQuant^®^ Analyser 10, Miltenyi Biotech Ltd., Surrey, UK).

For confocal microscopy studies, circular glass-coverslips (22 mm diameter, No. 1—0.13 to 0.17 mm thick) were placed in 6-well plates. Cells were seeded over the coverslip at a density of 150,000 cells per well and incubated overnight. Following this incubation period, cells were washed with D-PBS (2 mL, pH = 7.1) and MEM, devoid of FBS was added (1.5 mL) to each well. 4APPA-FAM^TM^-siRNA complexes (10 μg siRNA, 500 μL), prepared as described above for the FACS analyses were added to cells and the cells incubated for 6 h. Lipofectamine 2000^TM^-FAM^TM^-siRNA complexes were prepared as described above for the FACS analysis containing 3 μg siRNA in a 30 μL volume. Lipofectamine 2000 ^TM^ complexes served as a positive control while non-treated cells and cells treated with FAM^TM^-siRNA alone served as negative controls. At the end of the incubation period, the medium was removed and the cells washed three times with D-PBS (2 mL, pH = 7.1) to ensure all formulations had been removed. A fresh working solution was prepared (10 mL of 1× working solution from 1000× concentrated stain solution by adding 10 μL stain to 10 mL of PBS, pH = 7.4) of the plasma membrane stain (CellMask^TM^ Deep Red plasma membrane stain, fluorescence excitation/emission 649/666 nm, Invitrogen Life Technologies, UK). This plasma membrane stain solution was incubated with cells growing on coverslips at 37 °C for 3 min followed by three cycles of washing with D-PBS (2 mL, pH = 7.1). The cells were fixed using formalin (4% *w/v* Formaldehyde, pH = 6.9) for 10 min and then washed three times with D-PBS (2 mL, pH = 7.1) and a mounting anti-fade medium with built-in nuclear staining agent [Vectashield^®^ + DAPI (2-(4-amidinophenyl)-1H -indole-6-carboxamidine −1.5 mg mL^−1^), 10–20 μL] was applied to the coverslip. The samples were then mounted on a glass slide using mounting medium. Sample slides were stored in the fridge overnight in a light-protection box and the next day the slides were viewed using CLSM (Carl Zeiss, LSM 710 Confocal Microscope, Zeiss, Cambridge, UK).

### 2.5. In-Vivo Intranasal Brain Delivery

All animal experiments were conducted under the appropriate legislation (Animal Scientific Procedures Act 1986) and after approval by the local ethics committee: UCL Animal Welfare and Ethical Review Body under a licensed framework (UK Home Office Project Licence Number PPL 70/8224) Brain biodistribution of siRNA complexes was investigated using fluorescently labelled siRNA. Lyophilised Cy^TM^3-labelled GAPDH siRNA (Ambion, Life Technologies Ltd., Paisley, UK) was used to prepare 4APPA-Cy^3^-siRNA-GAPDH complexes at a nitrogen to phosphate ratio of 32. Complexes were prepared by adding Cy^3^–siRNA-GAPDH (1 mg mL^−1^, 0.04 mL, Cy^3^-siRNA-GAPDH) in sterile phosphate buffer (2 mM, pH = 6.0) to 4APPA polymer (40.5 mg mL^−1^, 0.05 mL, 4APPA) in sterile phosphate buffer (2 mM, pH = 6.0) at an N/P ratio of 32. The final siRNA concentration was 0.444 mg mL^−1^ in a final complex volume of 0.09 mL. The complexes (0.09 mL) were gently mixed and incubated at room temperature for 1 hr to ensure dynamic stability of the complex was reached. The tube was wrapped in aluminium foil to protect from light.

Male Sprague Dawley rats weighing between 220 and 260 g were used for the in vivo experiments. Animals were subjected to light anaesthesia using isoflurane (Forthane^®^, Abbott, UK) for 3–5 min, after which they were intranasally dosed using a 0.1 mL syringe attached to a flexible 1.5 cm length of tubing. Rats were either dosed with formulations (in phosphate buffer, 2 mM, pH = 6.0) containing: (a) Cy^3^-siRNA-GAPDH alone (1 mg mL^−1^, 20 μL), or (b) 4APPA-Cy^3^-siRNA-GAPDH complex (22.5 mg mL^−1^ 4APPA and 0.444 mg mL^−1^ siRNA in 0.045 mL). After dosing, the animals were placed on their backs (with heads pointing upwards to maximise residency times of exogenous substances in the olfactory area). The dose was administered intranasally into one nostril over 15–30 s. Animals were killed at the following time points: 5 min and 1 h after dosing. Animals were killed by an intraperitoneal injection of Euthatal^®^ (0.6 mL), followed by cervical dislocation. Brain and olfactory bulbs were harvested and snap frozen inside 50 mL tubes. All tissues were stored in −80 °C until analyses performed.

CLSM was used to visualise the localisation of the fluorescently labelled siRNA, in the brain and olfactory bulb tissues. The brain and olfactory bulbs were defrosted at room temperature. A small sample of tissue was cut from the surface of the olfactory bulb, the cerebral cortex and mid-brain. For confocal imaging, samples were placed on a glass slide, fixed with Formalin^®^ (4% *w*/*v* Formaldehyde) for 10 min followed by a phosphate buffer (2 mM, pH = 6.0) washing cycle and then mounted using anti-fade medium [Vectashield^®^ + DAPI (2-(4-amidinophenyl)-1H -indole-6-carboxamidine −1.5 mg mL^−1^), 10–20 μL]. The samples were covered by a coverslip and left in a fridge overnight in a light-protected box. The following day, slides were imaged using CLSM.

4APPA-SiRNA-ITCH was used to investigate protein down-regulation in vivo. 4APPA complexes with siRNA-ITCH were prepared at a nitrogen to phosphate ratio of 32 by adding siRNA-ITCH (2 mg mL^−1^, 0.04 mL) in sterile phosphate buffer (2 mM, pH = 6.0) to the 4APPA polymer (81 mg mL^−1^, 0.05 mL). The final siRNA concentration was 0.89 mg mL^−1^ in a final complex volume of 0.09 mL. The complexes were gently mixed and allowed to incubate at room temperature for 1 h to ensure dynamic stability of the complex was reached.

Groups (n = 5) of male Sprague Dawley rats weighing between 220 and 260 g were subjected to light anaesthesia using isoflurane (Forthane, Abbott, UK) for 3–5 min and were intranasally dosed using a 0.1 mL syringe attached to a flexible 1.5 cm length of tubing. Each animal was dosed twice a day and there were the following treatment groups (all dosed in phosphate buffer, 2 mM, pH = 6.0): siRNA-ITCH alone (1 mg mL^−1^, 40 μL), 4APPA-siRNA-ITCH (45 mg mL^−1^ and 0.89 mg mL^−1^, of 4APPA and siRNA, respectively, in an intranasal dose volume of 45 μL), 4APPA—scrambled siRNA (45 mg mL^−1^ and 0.89 mg mL^−1^, of 4APPA and siRNA, respectively, in an intranasal dose volume of 45 μL) and PBS (pH = 7.4, 40 μL). After dosing, the animals were placed on their backs (with heads pointing upwards to maximise the residence time of the dose within the olfactory area). The dose was administered intranasally into one nostril over 15–30 s. Animals were dosed twice daily for 3 days and killed 18 h after the sixth (last) dose was administered. Animals were killed by an intraperitoneal injection of Euthatal^®^ (0.6 mL), followed by cervical dislocation. Brain and Olfactory bulbs and whole brain were dissected, and snap frozen inside 50mL falcon tubes. All tissues were stored in −80 °C until analyses could be performed.

Brain and olfactory bulb tissue samples were defrosted, homogenised together (Dounce Tissue Grinder Complete All-Glass set, DWK Life Sciences GmbH, Mainz, Germany) on ice in 20 mL lysis buffer (Tissue Protein Extraction Reagent (T-PER) with phosphatase and protease inhibitors [Thermo Scientific Halt Protease Inhibitor Cocktail, ThermoFisher Scientific, Watford, UK (containing protease and phosphatase inhibitors and EDTA, at a ratio of 1 mL T-PER buffer to 0.01 mL of Halt Protease Inhibitor Cocktail)] for every 1g of tissue. Clean homogenisers were used for each sample to minimise crossover contamination. Once homogenised, tissues were centrifuged (14,400× *g*, 15 min, 4 °C) to pellet cell and tissue debris and the supernatant removed to new tubes. Protein concentration was determined using a micro-BCA assay kit according to manufacturer’s instructions (ThermoFisher Scientific, Watford, UK). Then, a protein aliquot of 60 μg was subjected to Western blot SDS-PAGE gel analysis (200 V for 50 min). At the end of the electrophoresis experiment, the gel was transferred to a nitrocellulose membrane. Membranes were washed and blocked with blocking reagent (5% *w*/*v* skimmed milk in PBS pH = 7.4, containing 0.1% *v*/*v* Tween 20) overnight at 4 °C to prevent non-specific binding. The membrane was washed five times over 30 min, with PBS containing Tween 20 (0.1% *v*/*v*) for 3 washes and PBS (pH = 7.4) for 2 washes. Western Blots were immunostained with primary antibodies for anti-actin protein (the house keeping gene, mouse monoclonal IgG1 to actin, 0.2 mg mL^−1^, 4 μL) and anti-ITCH protein (target gene, mouse monoclonal IgG1 to ITCH, 0.25 mg mL^−1^, 20 μL) all diluted in Tween 20 (0.1% *v*/*v* in PBS, 10 mL). Immunostaining was carried out for 2 hrs at room temperature; after which membranes were rinsed three times in PBS (pH = 7.4) containing Tween 20 (0.1% *v*/*v*) for 5 min each, followed by a further two washes in PBS (pH = 7.4) only for 5 min each.

Washings were followed by labelling with the secondary antibody conjugated to horseradish peroxidase (1 mg mL^−1^, 10 μL) diluted in Tween 20 (0.1% *v*/*v* in PBS, 10 mL, pH = 7.4) for 1 h. The five washing steps described above were repeated to remove labelling reagent residue and the blot was developed with SuperSignal westPICO chemiluminescent detection kit as per manufacturer’s instructions (Thermo Scientific, Watford, UK) and imaged using a UV camera.

Data were generated as gel blots images. Furthermore, the ITCH relative expression levels were normalised to the reference protein actin to correct for sample variation. The ratio of ITCH expression to actin expression was determined and ITCH expression was compared between samples. ITCH protein levels were expressed as down-regulation percentages by measuring the signal optical density (Western blot densitometry analysis) using a UV camera.

### 2.6. Statistics

Statistical analyses were carried out using one-way ANOVA, followed by post hoc tests. A *p*-value of <0.05 was considered to be statistically significant. Analyses were performed using OriginPro^®^ (version 8.6, OriginLab Corporation, Northampton, MA, USA) and Prism^®^ (version 6.0c). Mean values and standard deviations are reported unless otherwise stated.

## 3. Results

### 3.1. Synthesis of Tetra-O,O,O,O-[Poly(Ethyleneglycol-O-2-Ethyleneimine)-Graft-N-(3-Propylamine)-]-Pentaerythritol (4APPA) and Polyplex Formation

4APPA was synthesised in acceptable yield (30%), although future work is required to maximise this yield. Approximately one of the four amine groups in 4APEG-NH_2_ were derivatised, despite the fact that each mole of 4APEG-NH_2_ was reacted with 16 moles of 3-Boc-APBr. MALDI-TOF spectra are given in the Figure S1 of the Supplementary Information file. The low level of derivatisation was advantageous as a high level of amines moieties on molecules contributes to toxicity [16,20].

**Figure 2:**
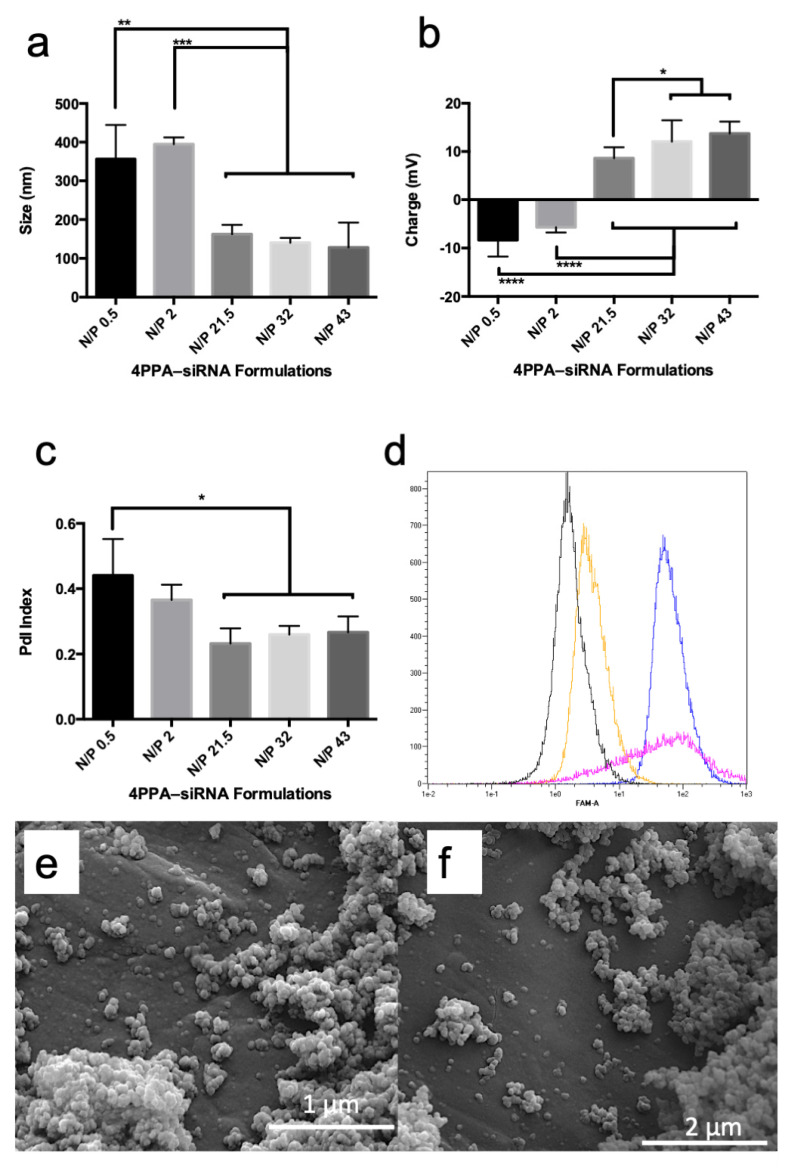
4APPA-siRNA polyplexes containing 20 μg mL^−1^ siRNA in phosphate buffer (2 mM, pH = 6.0) (**a**) polyplex z-average mean size, (**b**) polyplex zeta potential, (**c**) polyplex polydispersity, all particle characterisation data are shown as mean ± s.d. (n = 3 separate experiments, * = statistically significant difference *p* < 0.05, ** = statistically significant difference *p* < 0.01, *** = statistically significant difference *p* < 0.001, **** = statistically significant difference *p* < 0.0001), (**d**) fluorescent activated cell sorting histograms from the FAM channel following incubation of FAM™-siRNA with A431 cells for 6 h (black = cells alone, yellow = cells plus FAM™-siRNA alone, pink = cells plus FAM™-siRNa, Lipofectamine, blue = 4APPA-FAM™-siRNA, (**e**) SEM image of 4APPA-siRNA at an N/P ratio of 43, (**f**) SEM image of 4APPA-siRNA at an N/P ratio of 32.

4APPA forms polyplexes with siRNA at pH = 6.0 due to an electrostatic interaction between the protonated amine groups on 4APPA and anionic phosphate groups on the siRNA. Polyplexes were 200 nm in size, had a polydispersity of 0.2 and a positive zeta potential (>+10 mV) when the nitrogen to phosphate (N/P) ratio of 4APPA to siRNA was 21.5, 32 or 43 (Figure 2a–c). At lower N/P ratios (0.5 and 2) the polyplexes were negatively charged and presented with larger sizes indicating incomplete complexation with siRNA, the possibility of free siRNA in the complex mixture and particle aggregation. It is well known that a minimum N/P ratio is required for stable nucleic acid complexes to form and that a large proportion of nitrogen atoms on the nucleic acid complexing cationic polymer molecule are not engaged in the polyplex formation [21]. The positive zeta potential contributes to the colloidal stability of the polyplexes by preventing aggregation due to charge repulsion [22]. The polyplexes made with an N/P ratio of 32 or 43 present as spherical 100–200 nm objects when viewed using scanning electron microscopy (Figure 2).

### 3.2. Cell Cytotoxicity, Cell Uptake and In Vitro down Regulation

Prior to the cell transfection studies the cell cytotoxicity of the new carrier was measured in the A431 cell line using the MTT assay. Data in Table 1 demonstrate that 4APPA is significantly less toxic than Lipofectamine. Amine polymers used for transfection are typically comparatively toxic with the A431 cell line IC50 of the transfection agents—Generation 3 polypropylenimine dendrimer (DAB 16) [21] and poly(ethylenimine) 25 kDa [16] being 0.039 mg mL^−1^ and 0.0019 mg mL^−1^, respectively, and the presence of tertiary amines, in particular, in a chitosan based transfection agent made this agent more toxic [15]. Here, we show that limiting the amines in general has a profoundly significant effect on the cell cytotoxicity and we have produced a new design of transfection agent with amines limited to the periphery of a branched molecule and the more biocompatible poly(ethylene glycol) units comprising the molecular backbone (Figure 1). 4APPA was almost three orders of magnitude less toxic than Lipofectamine (Table 1) and its IC50 was similar to the starting material 4APEG-NH_2_.

We observed a similar biocompatibility advantage with tetra-O,O,O,O-[poly(ethyleneglycol-O-2-ethyleneimine)-graft-N-(2-ethylamine)-]-pentaerythritol (4-arm-PEG-ethylamine—4APEA), a 4APEG-NH_2_ derivative synthesised for intravenous gene delivery [23]. 4APEA had an IC50 of 4.76 ± 0.27 mg in the A431 cell line.

In order to explore cell uptake of the polyplexes, cells were incubated with 4APPA-FAM™-siRNA (N/P ratio = 32) and cell association explored with FACS analyses and with CLSM. Figure 2d shows that when cells are incubated with 4APPA-FAM™-siRNA for 6 h, FAM™-siRNA is associated with the cells following incubation and this association is higher than when FAM™-siRNA alone is incubated with the cells and the association is to a similar extent to Lipofectamine-FAM™-siRNA. The FACS data also reveal information on cell viability, as an examination of the forward scatter (FSC) histogram (Supplementary Information Figure S2) shows that while the control, FAM™-siRNA alone and 4APPA-FAM™-siRNA cells have a strong fluorescence intensity (an indication of a minimum of dead cells and cell debris), cells treated with Lipofectamine-FAM™-siRNA reveal a lower fluorescence intensity which is indicative of a reduced level of viable cells. The toxicity of Lipofectamine indicated in Table 1 is in evidence with these data.

**Figure 3:**
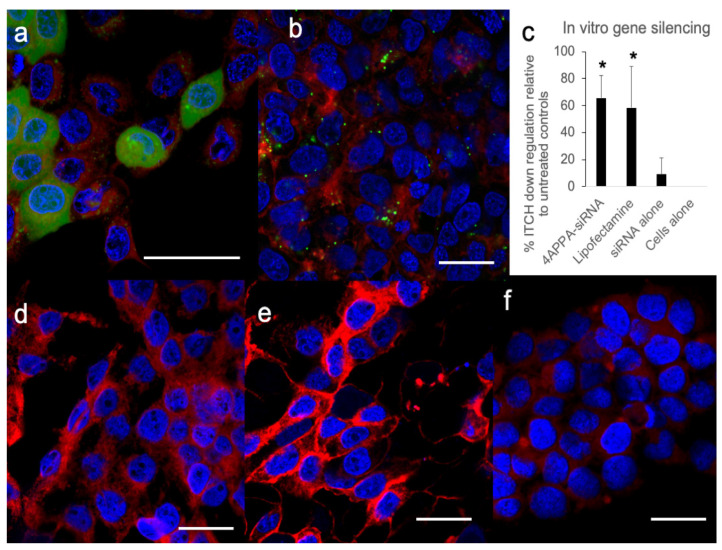
CLSM images (**a**,**b**,**d**–**f**) and in vitro gene silencing using siRNA (**c**) in A431 cells. (**a**,**b**,**d**–**f**)—A431 cells (seeded at 150,000 cells per well) with the cell nucleus stained blue (DAPI), cell cytoplasm stained red (CellMask™) and FAM™-siRNA stained green: (**a**) cells were treated with 4APPA-FAM™-siRNA (20 μg mL^−1^ siRNA, 500 μL) for 6 h, (**b**) cells were treated with Lipofectamine-FAM™-siRNA (100 μg mL^−1^ siRNA, 30 μL) according to the manufacturer’s instructions, (**d**) cells were treated with PBS, (**e**) cells were treated with FAM™-siRNA alone (20 μg mL^−1^ siRNA, 500 μL), (**f**) untreated control cells. Bar = 20 μm. Data showing separate images from the FAM, DAPI and CellMask channels may be found in Figures S3–S7 in the Supplementary Information file. (**c**) Western blot densitometry analysis of ITCH gene silencing showing percentage ITCH down regulation when compared to siRNA alone (mean ± s.d., n = 5 and representative of 3 separate experiments) in A431 cells (seeded at 400,000 cells per flask) incubated for 6 h with 4APPA-siRNA (20 μg mL^−1^ siRNA, 500 μL, N, P ratio = 32 in phosphate buffer—2 mM, pH = 6.0), Lipofectamine 2000™-siRNA (100 μg mL^−1^ siRNA, 30 μL) prepared according to the manufacturer’s instructions, siRNA alone (20 μg mL^−1^ siRNA, 500 μL), Western blot gels may be found in Figure S8 in the Supplementary Information file. * = statistically significant difference *p* < 0.05.

In order to distinguish between cell association and cell uptake various FAM™-siRNA formulations were incubated with the cells and examined by CLSM (Figure 3).

CLSM images (Figure 3) show that 4APPA enabled the uptake of fluorescently labelled siRNA into A431 cells after incubation for 6 h. Both punctate and diffuse green fluorescence, originating from intracellular FAM™-siRNA can be seen in Figure 3a, while cells treated with naked siRNA (Figure 3e) showed no green fluorescence at all within the cells. Cells treated with the positive control vector—Lipofectamine—also show punctate green fluorescence signals originating from FAM™-siRNA from within the cells (Figure 3b), while cells treated with PBS and untreated cells, and show no green fluorescence (Figure 3d and Figure 3f respectively). The data complement the FACS data in that these data show that there is 4APPA facilitated cell uptake of siRNA. The punctate nature of the image indicates that endosomal uptake mechanisms are likely to be responsible for the cellular uptake [24]. We speculate that the more diffuse green fluorescence seen in Figure 3a could indicate an exit of siRNA from the endosome and its location within the cytoplasm. The vast majority of the fluorescence appears to be in the perinuclear and cytoplasm regions of the cell (Figure 3a,b).

ITCH gene silencing (Figure 3c) was observed with 4APPA-siRNA polyplexes in the A431 cell line (Figure 3c). There was 65% less protein expression with the 4APPA polyplexes when compared to siRNA alone. Additionally, incubation of the cells with Lipofectamine-siRNA also resulted in 58% gene silencing. We have established that 4APPA polyplexes deliver siRNA into cells and this results in gene silencing in vitro.

### 3.3. In Vivo siRNA Delivery and In Vivo Brain Gene Silencing

**Figure 4:**
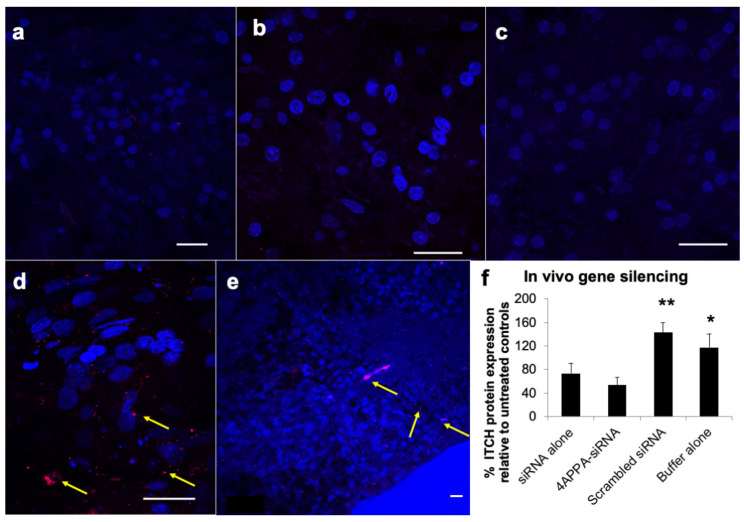
CLSM images (**a**–**e**) with the nucleus stained blue with DAPI and Cy^3^-siRNA-GAPDH stained red–arrowed and in vivo gene silencing (**f**) in rat brain tissue. Bar = 20 μm; All formulations were dosed in phosphate buffer (2 mM, pH = 6.0): (**a**) rat olfactory bulb 5 min after the intranasal delivery of Cy^3^-siRNA-GAPDH alone (1 mg mL^−1^, 20 μL), (**b**) rat cerebral cortex 1 h after the intranasal administration of Cy^3^-siRNA-GAPDH alone (1 mg mL^−1^, 20 μL), (**c**) rat mid brain 1 h after the intranasal administration of Cy^3^-siRNA-GAPDH alone (1 mg mL^−1^, 20 μL), (**d**) rat olfactory bulb 5 min after the intranasal administration of 4APPA-Cy^3^-siRNA-GAPDH polyplexes (0.444 mg mL^−1^ siRNA, 45 μL, N, P ratio = 32), (**e**) rat cerebral cortex 1 h after the intranasal administration of 4APPA-Cy^3^-siRNA-GAPDH polyplexes (0.444 mg mL^−1^ siRNA, 45 μL, N, P ratio = 32), (**f**) percentage ITCH protein expression in rat brains (mean ± s.d., n = 5 and representative of 2 separate experiments) following the nasal administration of 4APPA-siRNA (0.9 mg mL^−1^ siRNA, 45 μL, N, P ratio = 32 in phosphate buffer—2 mM), siRNA (0.9 mg mL^−1^ siRNA, 45 μL), scrambled siRNA (0.89 mg mL^−1^ siRNA, 45 μL) and phosphate buffer (2 mM) to male Sprague Dawley rats dosed twice daily for 3 days and killed 18 h after the last dose. * = statistically significantly different when compared to all other formulations (*p* < 0.05), ** = statistically significantly different when compared to all other formulations (*p* < 0.0001).

The delivery of siRNA to the brain tissue was analysed using fluorescently labelled siRNA (Cy^3^-siRNA-GAPDH, Figure 4). The siRNA fluorescent label was found in the olfactory bulb at 5 min after dosing, the cerebral cortex 1 h after dosing and to a lesser extent in the mid-brain 1 h after dosing, when the 4APPA-Cy^3^-siRNA-GAPDH polyplexes were administered intranasally to a healthy rat model (Figure 4d,e and Figure S9 in the Supplementary Information file). Examination of the olfactory bulb 5 min after dosing and the cerebral cortex and mid-brain 1 h after dosing revealed the ability of 4APPA to facilitate delivery of siRNA on intranasal dosing to the distal brain regions, such as the cerebral cortex and, to a lesser extent, in the mid brain (Figure 4e and Figure S9). The fluorescent signal was found to a far lesser extent in the olfactory bulb when Cy^3^-siRNA-GAPDH alone was administered intranasally (Figure 4a) and was very difficult to detect in the cerebral cortex and mid brain when siRNA alone was administered intranasally (Figure 4b,c). siRNA gene silencing was also measured in the brain and olfactory bulb tissue and 4APPA-siRNA-ITCH complexes resulted in reduced levels of ITCH (54 ± 13% ITCH remaining) in the olfactory bulb and brain tissue when compared to untreated controls (Figure 4f). It is clear that the intranasal delivery of siRNA bound to 4APPA polyplexes resulted in gene silencing. Interestingly, modest brain gene silencing (73 ± 17% ITCH remaining) was also found on the intranasal administration of siRNA alone (Figure 4f). The high level of siRNA fluorescent signal found in the olfactory bulb 5 min after dosing indicates that siRNA travels through the olfactory bulb and into the deeper brain, and this transport is enhanced using 4APPA polyplexes.

## 4. Discussion

The possibility of using gene silencing to treat neurological diseases of the brain is attractive. Gene silencing occurs when siRNA mediated degradation of mRNA leads to reduced protein expression [25,26]. Approved siRNA therapeutics are now available for the treatment of hereditary amyloidogenic transthyretin amyloidosis with polyneuropathy in adults (patisiran) and acute hepatic porphyria in adults (givosiran) [27]. However no approved siRNA therapeutics exist for the treatment of any neurological diseases. Neurological diseases originating in the brain are often chronic and require multiple applications of the therapeutic to the brain, making the biocompatibility of any carriers especially important. Delivering the siRNA to the brain tissue is still challenging as intravenous methods are hampered by the blood brain barrier; a barrier comprising specialised endothelial cells, degradative enzymes and efflux pumps [28]; all limiting the access of therapeutics to the brain parenchyma. The use of specific intraarterially injected AAVs has shown promise for brain gene replacement therapy [4] but these technologies have not been shown to be applicable to gene silencing. For chronic therapeutic gene silencing, we require a relatively biocompatible formulation which may be applied repeatedly via a non-invasive route.

We have previously reported the synthesis of a relatively biocompatible gene delivery polymer—4APEA (IC50 in the A431 cell line = 4.76 ± 0.27 mg mL^−1^) that successfully delivered genes to tumours on intravenous administration [23]. Here, we show, for the first time, that a relatively biocompatible nucleic acid transfer molecule: 4APPA (IC50 = 13.97 ± 6 mg mL^−1^ in the A431 cell line—Table 1) that is 3–4 orders of magnitude less cytotoxic than both Lipofectamine (Table 1) and 25 kDa poly(ethylenimine) [16], is able to complex with siRNA to form 150 nm positively charged complexes at an siRNA to 4APPA weight ratio of ~1:40, with the siRNA complexes being taken up by cells in culture and effecting gene silencing of the demonstrator ITCH gene (Figure 2 and Figure 3). 4APPA, a derivative of 4APEG-NH_2_, in which an average of only one in 4 amine groups are derivatised with propylamine. This low level of derivatisation was deliberately chosen in order to engineer a biocompatible vector. We know from previous work that limiting the level of tertiary amines in particular was associated with less cytotoxicity of a polymer siRNA vector [15] and that 4APEG-NH2 is a suitable synthetic scaffold from which to construct gene delivery vectors [23] and here we show that a low level of amine moieties on 4APPA is associated with good cell biocompatibility (Table 1).

When siRNA-4APPA polyplexes were administered via the nose to brain route, 4APPA delivered siRNA to the olfactory bulb and frontal cortex (Figure 4) and mid-brain (Figure S9) and resulted in specific brain gene silencing (Figure 4). Qualitative data reveal fluorescent siRNA is seen in the olfactory bulb 5 min after dosing (Figure 4d), indicating that this is the route of entry of siRNA into the brain. We have previously shown siRNA may be delivered to olfactory bulb neurons on intranasal dosing of siRNA polyplexes [15]. The level of gene silencing seen with the 4APPA polyplexes (Figure 4), when compared to naked siRNA indicates that this positively charged polyplex is able to enhance the transport of siRNA within the brain and facilitate entry of siRNA into brain cells. The dose of siRNA used in the current study was low at 0.15 mg kg^−1^ in the healthy rat model with each animal receiving a total of 240 μg siRNA over 6 doses to achieve 46% ITCH gene silencing. Other intranasal studies do not provide enough detail to determine the actual dose of siRNA used over multiple dose events. However, the level of gene silencing obtained in the current study compares favourably with other studies where siRNA (at least 5.8 nmol or 77 μg siRNA) in a chitosan–mangafodipir formulation; on being administered twice daily for 5 days to a transgenic mouse model (a tenth of the weight of rat model) achieved 50% huntingtin gene silencing [13]. Further studies report just a maximum of 40% gene silencing in a rat model using a PAMAM dendrimer derivative [14] and 50% gene silencing in a mouse model using poly(ethylenimine) [12].

Transport of particles to the brain via the olfactory bulb are hypothesised to proceed via transport through olfactory neurons which is then followed by a wider distribution to the brain being via perivascular transport [9,29]. We observe fluorescently labelled siRNA in the cerebral cortex following intranasal administration of fluorescently labelled siRNA-4APPA and data from others suggests that gene silencing in the cerebral cortex following administration of siRNA nanoparticles appears prior to gene silencing in other regions [13]. We speculate that it should be possible to target diseases manifesting in the cerebral cortex and thus target conditions affecting cognition.

The ITCH gene is present in rat brains, but, crucially, is present in human tumours and presents a potential context-dependent therapeutic target [30]. In glioblastoma, an aggressive form of brain cancer, E3 ligases are known to regulate important cellular functions including pro-survival signalling [31]. Down regulation of ITCH also potentially contributes to a tumouricidal response via the de-repression of the P73 pathway [32]. As down regulation of ITCH is observed with our 4APPA-siRNA-ITCH formulation it is possible that this approach could also be used to treat intracranial tumours.

The nose to brain route appears to be a promising route for siRNA delivery. However there are key limitations with this route, such as the dose which is limited to 30 mg of solid or 100 μL of liquid [9]. Additionally, to protect the nasal passages, nasal drug delivery systems should be non-irritant and non-cytotoxic at the doses given. In contrast to our previous publication [11], the nasal irritancy of the formulation was not formally studied in this manuscript.

## 5. Conclusions

Our data demonstrate that brain gene silencing may be achieved with the intranasal administration of siRNA polyplexes based on a new relatively biocompatible delivery system, based on star-shaped poly(ethylene glycol) 4APPA.

## Figures and Tables

**Table 1:** In vitro cytotoxicity (IC50) in the A-431 cell line.

Transfection Reagent	IC50 mg mL^−1^ (Mean ± s.d.), A-431 Cells
4APEG-NH_2_	18.61 ± 9.0
4APPA	13.92 ± 6.0
Lipofectamine^®^ 2000	0.033 ± 0.04 *

* = statistically significantly different from all other transfection agents *p* < 0.05.

## Data Availability

The raw/processed data required to reproduce these findings cannot be shared at this time as the data also form part of an ongoing study.

## Supplementary Materials

The following supporting information can be downloaded at: https://www.mdpi.com/article/10.3390/biomedicines10092182/s1. Figure S1: 4APPA MALDI-TOF spectrum; Figure S2: Fluorescent activated cell sorting histograms from the Forward Scatter (FSC) channel following incubation of FAM™-siRNA with A431 cells for 6 h (black = cells alone, yellow = cells plus FAM™-siRNA alone, pink = cells plus FAM™-siRNa, Lipofectamine, blue = 4APPA-FAM™-siRNA; Figure S3: CLSM images of A431 cells (seeded at 150,000 cells per well) with the cell nucleus stained blue (DAPI), cell cytoplasm stained red (CellMask™) and FAM™-siRNA stained green, cells were treated with 4APPA-FAM™-siRNA (20 μg mL^−1^ siRNA, 500 μL) for 6 h: (a) DAPI channel, (b) FAM channel, (c) cell mask channel, (d) merged image. Bar = 20 μm; Figure S4: CLSM images of A431 cells (seeded at 150,000 cells per well) with the cell nucleus stained blue (DAPI), cell cytoplasm stained red (CellMask™) and FAM™-siRNA stained green, cells were treated with 4APPA-FAM™-siRNA (20 μg mL^−1^ siRNA, 500 μL) for 6 h: (a) DAPI channel, (b) FAM channel, (c) cell mask channel, d) merged image. Bar = 20 μm; Figure S5: CLSM images of A431 cells (seeded at 150,000 cells per well) with the cell nucleus stained blue (DAPI), cell cytoplasm stained red (CellMask™) and FAM™-siRNA stained green, cells were treated with Lipofectamine-FAM™-siRNA (100 μg mL^−1^ siRNA, 30 μL made according to the manufacturer’s instructions) for 6 h: (a) DAPI channel, (b) FAM channel, (c) cell mask channel, (d) merged image. Bar = 20 μm.; Figure S6: CLSM images of A431 cells (seeded at 150,000 cells per well) with the cell nucleus stained blue (DAPI), cell cytoplasm stained red (CellMask™) and FAM™-siRNA stained green, cells were treated with PBS (500 μL) for 6 h: (a) DAPI channel, (b) FAM channel, (c) cell mask channel, d) merged image. Bar = 20 μm; Figure S7: CLSM images of A431 cells (seeded at 150,000 cells per well) with the cell nucleus stained blue (DAPI), cell cytoplasm stained red (CellMask™) and FAM™-siRNA stained green, cells were treated with FAM™-siRNA alone (20 μg mL^−1^ siRNA, 500 μL) for 6 h: (a) DAPI channel, (b) FAM channel, (c) cell mask channel, (d) merged image. Bar = 20 μm; Figure S8: Western blot analysis of ITCH gene silencing when compared to siRNA alone (n = 5 replicates and representative gel of 3 separate experiments) in A431 cells (seeded at 400,000 cells per flask) incubated for 6 h with scrambled siRNA (20 μg mL^−1^ siRNA, 500 μL in phosphate buffer—2 mM), siRNA-ITCH alone (20 μg mL^−1^ siRNA, 500 μL, in phosphate buffer—2 mM), 4APPA-siRNA (20 μg mL^−1^ siRNA, 500 μL, N, P ratio = 32 in phosphate buffer—2 mM), 4APPA-siRNA (20 μg mL^−1^ siRNA, 500 μL, N, P ratio = 43 in phosphate buffer—2 mM), Lipofectamine 2000™-siRNA (100 μg mL^−1^ siRNA, 30 μL) prepared according to the manufacturer’s instruction; Figure S9: CLSM image (with the nucleus stained blue with DAPI and Cy^3^-siRNA-GAPDH stained red) of the rat mid brain 1 h after the intranasal administration of 4APPA-Cy^3^-siRNA-GAPDH polyplexes (0.444 mg mL^−1^ siRNA, 45 μL, N, P ratio = 32). Arrows show sparse fluorescent signals originating from the fluorescent siRNA

## References

[1] Thomas S.J., Moreira E.D., Kitchin N., Absalon J., Gurtman A., Lockhart S., Perez J.L., Perez Marc G., Polack F.P., Zerbini C. (2021). Safety and Efficacy of the BNT162b2 mRNA COVID-19 Vaccine through 6 Months. N. Engl. J. Med..

[2] Adams D., Gonzalez-Duarte A., O’Riordan W.D., Yang C.C., Ueda M., Kristen A.V., Tournev I., Schmidt H.H., Coelho T., Berk J.L. (2018). Patisiran, an RNAi Therapeutic, for Hereditary Transthyretin Amyloidosis. N. Engl. J. Med..

[3] Stevens D., Claborn M.K., Gildon B.L., Kessler T.L., Walker C. (2020). Onasemnogene Abeparvovec-xioi: Gene Therapy for Spinal Muscular Atrophy. Ann. Pharmacother..

[4] Yoon S.Y., Hunter J.E., Chawla S., Clarke D.L., Molony C., O’Donnell P.A., Bagel J.H., Kumar M., Poptani H., Vite C.H. (2020). Global CNS correction in a large brain model of human alpha-mannosidosis by intravascular gene therapy. Brain.

[5] Mazza M., Hadjidemetriou M., de Lazaro I., Bussy C., Kostarelos K. (2015). Peptide nanofiber complexes with siRNA for deep brain gene silencing by stereotactic neurosurgery. ACS Nano.

[6] Osborn M.F., Coles A.H., Golebiowski D., Echeverria D., Moazami M.P., Watts J.K., Sena-Esteves M., Khvorova A. (2018). Efficient Gene Silencing in Brain Tumors with Hydrophobically Modified siRNAs. Mol. Cancer Ther..

[7] Alterman J.F., Hall L.M., Coles A.H., Hassler M.R., Didiot M.-C., Chase K., Abraham J., Sottosanti E., Johnson E., Sapp E. (2015). Hydrophobically Modified siRNAs Silence Huntingtin mRNA in Primary Neurons and Mouse Brain. Mol. Ther.-Nucleic Acids.

[8] Godinho B., Henninger N., Bouley J., Alterman J.F., Haraszti R.A., Gilbert J.W., Sapp E., Coles A.H., Biscans A., Nikan M. (2018). Transvascular Delivery of Hydrophobically Modified siRNAs: Gene Silencing in the Rat Brain upon Disruption of the Blood-Brain Barrier. Mol. Ther..

[9] Wang Z., Xiong G., Tsang W.C., Schatzlein A.G., Uchegbu I.F. (2019). Nose-to-Brain Delivery. J. Pharmacol. Exp. Ther..

[10] Chapman C.D., Frey W.H., Craft S., Danielyan L., Hallschmid M., Schioth H.B., Benedict C. (2013). Intranasal treatment of central nervous system dysfunction in humans. Pharm. Res..

[11] Godfrey L., Iannitelli A., Garrett N.L., Moger J., Imbert I., King T., Porreca F., Soundararajan R., Lalatsa A., Schatzlein A.G. (2017). Nanoparticulate peptide delivery exclusively to the brain produces tolerance free analgesia. J. Control. Release.

[12] Rodriguez M., Lapierre J., Ojha C.R., Kaushik A., Batrakova E., Kashanchi F., Dever S.M., Nair M., El-Hage N. (2017). Intranasal drug delivery of small interfering RNA targeting Beclin1 encapsulated with polyethylenimine (PEI) in mouse brain to achieve HIV attenuation. Sci. Rep..

[13] Sava V., Fihurka O., Khvorova A., Sanchez-Ramos J. (2020). Enriched chitosan nanoparticles loaded with siRNA are effective in lowering Huntington’s disease gene expression following intranasal administration. Nanomed. Nanotechnol. Biol. Med..

[14] Kim I.D., Shin J.H., Lee H.K., Jin Y.C., Lee J.K. (2012). Intranasal delivery of HMGB1-binding heptamer peptide confers a robust neuroprotection in the postischemic brain. Neurosci. Lett..

[15] Simao Carlos M.I., Zheng K., Garrett N., Arifin N., Workman D.G., Kubajewska I., Halwani A.A., Moger J., Zhang Q., Schatzlein A.G. (2017). Limiting the level of tertiary amines on polyamines leads to biocompatible nucleic acid vectors. Int. J. Pharm..

[16] Brownlie A., Uchegbu I.F., Schatzlein A.G. (2004). PEI-based vesicle-polymer hybrid gene delivery system with improved biocompatibility. Int. J. Pharm..

[17] Kim I.D., Lim C.M., Kim J.B., Nam H.Y., Nam K., Kim S.W., Park J.S., Lee J.K. (2010). Neuroprotection by biodegradable PAMAM ester (e-PAM-R)-mediated HMGB1 siRNA delivery in primary cortical cultures and in the postischemic brain. J. Control. Release.

[18] Regge D., Cirillo S., Macera G. (2009). Mangafodipir trisodium: Review of its use as an injectable contrast medium for magnetic resonance imaging. Rep. Med. Imaging.

[19] Treinen K.A., Gray T.J., Blazak W.F. (1995). Developmental toxicity of mangafodipir trisodium and manganese chloride in Sprague-Dawley rats. Teratology.

[20] Malik N., Wiwattanapatapee R., Klopsch R., Lorenz K., Frey H., Weener J.W., Meijer E.W., Paulus W., Duncan R. (2000). Dendrimers: Relationship between structure and biocompatibility in vitro, and preliminary studies on the biodistribution of I- 125-labelled polyamidoamine dendrimers in vivo. J. Control. Release.

[21] Zinselmeyer B.H., Mackay S.P., Schatzlein A.G., Uchegbu I.F. (2002). The lower-generation polypropylenimine dendrimers are effective gene-transfer agents. Pharm. Res..

[22] Florence A.T., Attwood D. (2006). Physicochemical Principles of Pharmacy.

[23] Iemsam-Arng J., Kong X., Schätzlein A.G., Uchegbu I.F. (2014). Star Shaped Poly(ethylene glycols) Yield Biocompatible Gene Delivery Systems. Pharm. Nanotechnol..

[24] Rennick J.J., Johnston A.P.R., Parton R.G. (2021). Key principles and methods for studying the endocytosis of biological and nanoparticle therapeutics. Nat. Nanotechnol..

[25] Hutvagner G., Simard M.J., Mello C.C., Zamore P.D. (2004). Sequence-specific inhibition of small RNA function. PLoS Biol..

[26] Caplen N.J., Parrish S., Imani F., Fire A., Morgan R.A. (2001). Specific inhibition of gene expression by small double-stranded RNAs in invertebrate and vertebrate systems. Proc. Natl. Acad. Sci. USA.

[27] Hu B., Zhong L., Weng Y., Peng L., Huang Y., Zhao Y., Liang X.-J. (2020). Therapeutic siRNA: State of the art. Signal Transduct. Target. Ther..

[28] Lalatsa A., Schätzlein A.G., Uchegbu I.F., MurrayMoo-Young M., Butler M., Webb C., Moreira A., Grodzinski B., Cui Z. (2011). Drug delivery across the blood brain barrier. Comprehensive Biotechnology.

[29] Lochhead J.J., Wolak D.J., Pizzo M.E., Thorne R.G. (2015). Rapid transport within cerebral perivascular spaces underlies widespread tracer distribution in the brain after intranasal administration. J. Cereb. Blood Flow Metab..

[30] Yin Q., Wyatt C.J., Han T., Smalley K.S.M., Wan L. (2020). ITCH as a potential therapeutic target in human cancers. Semin. Cancer Biol..

[31] Humphreys L.M., Smith P., Chen Z., Fouad S., D’Angiolella V. (2021). The role of E3 ubiquitin ligases in the development and progression of glioblastoma. Cell Death Differ..

[32] de la Fuente M., Jones M.C., Santander-Ortega M.J., Mirenska A., Marimuthu P., Uchegbu I., Schatzlein A. (2015). A nano-enabled cancer-specific ITCH RNAi chemotherapy booster for pancreatic cancer. Nanomed. Nanotechnol. Biol. Med..

