# Supplementary material for: Brain Gene Silencing with Cationic Amino-Capped Poly(ethylene glycol) Polyplexes
Source: Biomedicines. 2022 Sep 3;10(9):2182. doi: 10.3390/biomedicines10092182 (PMC9496157; doi:10.3390/biomedicines10092182)
Supplement: Supplementary file 1 [file biomedicines-10-02182-s001.zip › biomedicines-1862105-supplementary.pdf]

Supplementary Information

Brain Gene Silencing with Cationic Amino-Capped Poly(Ethylene Glycol) Polyplexes

Abdullah A. Alamoudi [1,2], Paula A. Méndez [1,3], David Workman [1], Andreas G. Schätzlein [1,4] and Ijeoma F. Uchegbu [1,4,*]

[1] UCL School of Pharmacy, 29-39 Brunswick Square, London WC1N 1AX, UK
[2] Department of Pharmaceutics, Faculty of Pharmacy, King Abdulaziz University, Jeddah 21589, Saudi Arabia
[3] Materials Science Research Group, Institute of Chemistry, Faculty of Exact and Natural Sciences, University of Antioquia, Calle 70 N° 52-21, A.A 1226, Medellín 050010, Colombia
[4] Nanomerics Ltd., 2 London Wall Place, London EC2Y 5AU, UK

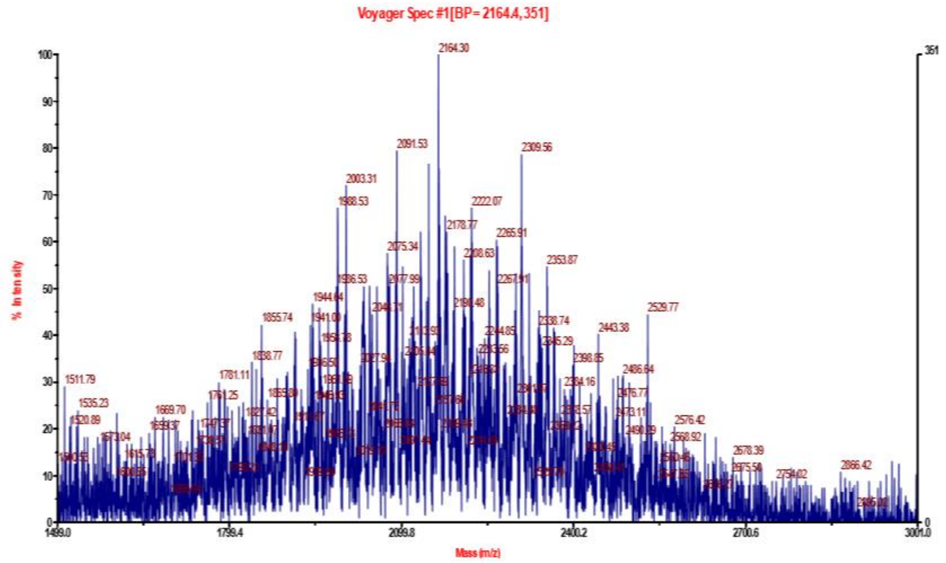

Figure S1. 4APPA MALDI-TOF spectrum

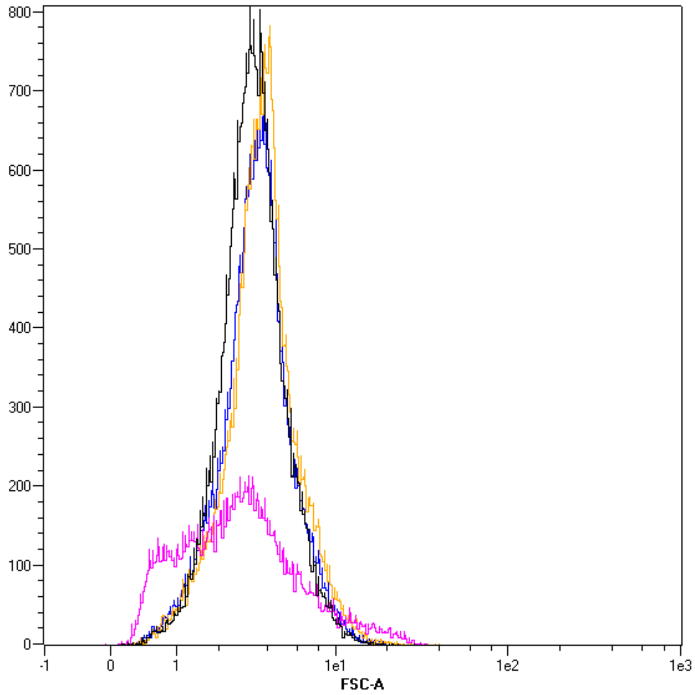

Figure S2: Fluorescent activated cell sorting histograms from the Forward Scatter (FSC) channel following incubation of FAM™-siRNA with A431 cells for 6 hours (black = cells alone, yellow = cells plus FAM™-siRNA alone, pink = cells plus FAM™-siRNa , Lipofectamine, blue = 4APPA – FAM™-siRNA

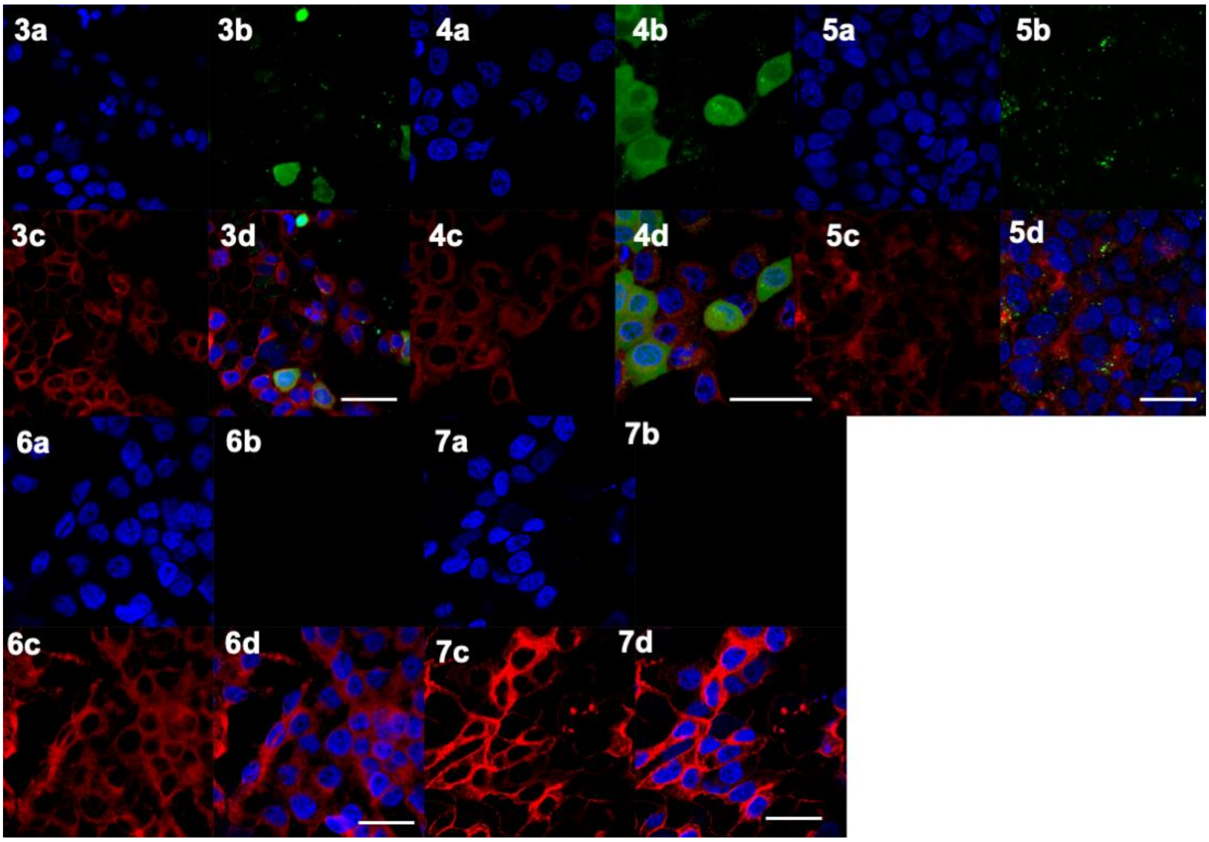

Figure S3: CLSM images of A431 cells (seeded at 150,000 cells per well) with the cell nucleus stained blue (DAPI), cell cytoplasm stained red (CellMask™) and FAM™-siRNA stained green, cells were treated with 4APPA - FAM™-siRNA (20 μg mL^{-1} siRNA, 500 μL) for 6 hours: a) DAPI channel, b) FAM channel, c) cell mask channel, d) merged image. Bar = 20 μm.

Figure S4: CLSM images of A431 cells (seeded at 150,000 cells per well) with the cell nucleus stained blue (DAPI), cell cytoplasm stained red (CellMask™) and FAM™-siRNA stained green, cells were treated with 4APPA - FAM™-siRNA (20 μg mL^{-1} siRNA, 500 μL) for 6 hours: a) DAPI channel, b) FAM channel, c) cell mask channel, d) merged image. Bar = 20 μm.

Figure S5: CLSM images of A431 cells (seeded at 150,000 cells per well) with the cell nucleus stained blue (DAPI), cell cytoplasm stained red (CellMask™) and FAM™-siRNA stained

green, cells were treated with Lipofectamine - FAM™-siRNA (100 μg mL^{-1} siRNA, 30 μL, made according to the manufacturer's instructions) for 6 hours: a) DAPI channel, b) FAM channel, c) cell mask channel, d) merged image. Bar = 20 μm.

Figure S6: CLSM images of A431 cells (seeded at 150,000 cells per well) with the cell nucleus stained blue (DAPI), cell cytoplasm stained red (CellMask™) and FAM™-siRNA stained green, cells were treated with PBS (500 μL) for 6 hours: a) DAPI channel, b) FAM channel, c) cell mask channel, d) merged image. Bar = 20 μm.

Figure S7: CLSM images of A431 cells (seeded at 150,000 cells per well) with the cell nucleus stained blue (DAPI), cell cytoplasm stained red (CellMask™) and FAM™-siRNA stained green, cells were treated with FAM™-siRNA alone (20 μg mL^{-1} siRNA, 500 μL) for 6 hours: a) DAPI channel, b) FAM channel, c) cell mask channel, d) merged image. Bar = 20 μm.

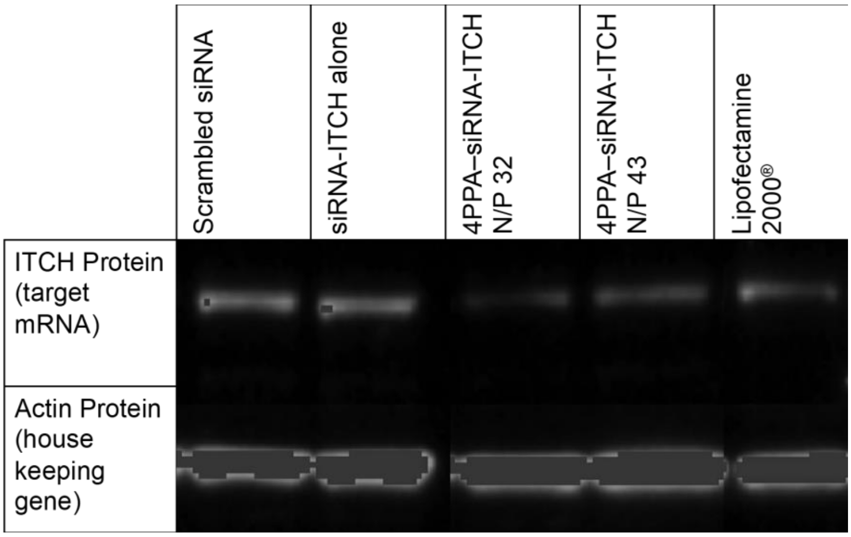

Figure S8: Western blot analysis of ITCH gene silencing when compared to siRNA alone (n = 5 replicates and representative gel of 3 separate experiments) in A431 cells (seeded at 400,000 cells per flask) incubated for 6 hours with scrambled siRNA (20 μg mL^{-1} siRNA, 500 μL in phosphate buffer - 2 mM), siRNA-ITCH alone (20 μg mL^{-1} siRNA, 500 μL, in phosphate

buffer - 2 mM), 4APPA-siRNA (20 μg mL^{-1} siRNA, 500 μL, N, P ratio = 32 in phosphate buffer - 2 mM), 4APPA-siRNA (20 μg mL^{-1} siRNA, 500 μL, N, P ratio = 43 in phosphate buffer - 2 mM), Lipofectamine 2000™-siRNA (100 μg mL^{-1} siRNA, 30 μL) prepared according to the manufacturer's instructions.

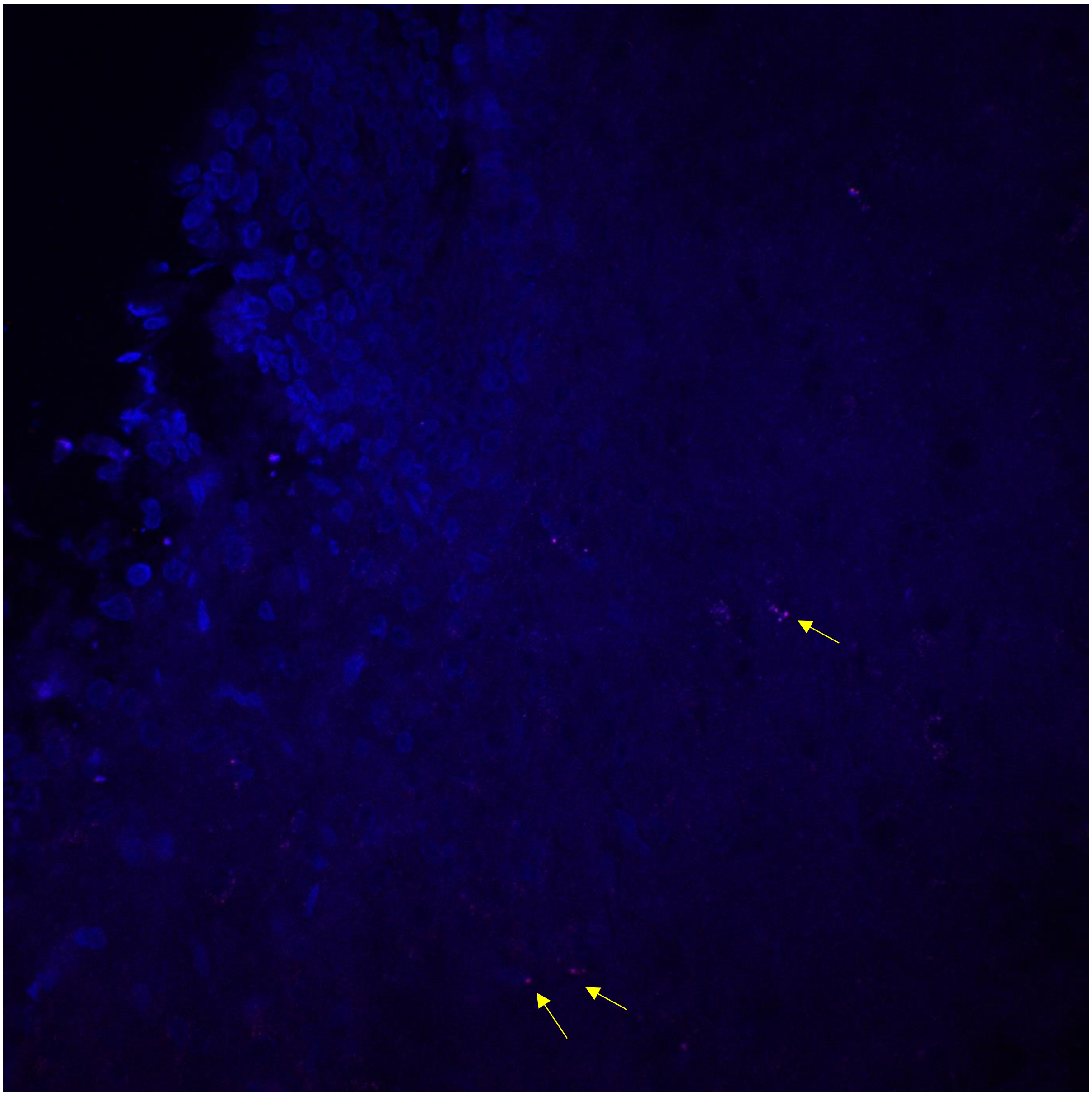

Figure S9: CLSM image (with the nucleus stained blue with DAPI and Cy^3-siRNA-GAPDH stained red) of the rat mid brain 1 hour after the intranasal administration of 4APPA- Cy^3-

siRNA-GAPDH polyplexes (0.444 mg mL^{-1} siRNA, 45 μL, N, P ratio = 32). Arrows show sparse fluorescent signals originating from the fluorescent siRNA.
